# A Kalman-Filter-Based Common Algorithm Approach for Object Detection in Surgery Scene to Assist Surgeon's Situation Awareness in Robot-Assisted Laparoscopic Surgery

**DOI:** 10.1155/2018/8079713

**Published:** 2018-05-02

**Authors:** Jiwon Ryu, Youngjin Moon, Jaesoon Choi, Hee Chan Kim

**Affiliations:** ^1^Department of Biomedical Engineering, Seoul National University, Seoul, Republic of Korea; ^2^Biomedical Engineering Research Center, Department of Convergence Medicine, Asan Medical Center, University of Ulsan College of Medicine, Seoul, Republic of Korea; ^3^Department of Biomedical Engineering, Asan Medical Center, University of Ulsan College of Medicine, Seoul, Republic of Korea; ^4^Department of Biomedical Engineering, College of Medicine, Institute of Medical and Biological Engineering, Medical Research Center, Seoul National University, Seoul, Republic of Korea

## Abstract

Although the use of the surgical robot is rapidly expanding for various medical treatments, there still exist safety issues and concerns about robot-assisted surgeries due to limited vision through a laparoscope, which may cause compromised situation awareness and surgical errors requiring rapid emergency conversion to open surgery. To assist surgeon's situation awareness and preventive emergency response, this study proposes situation information guidance through a vision-based common algorithm architecture for automatic detection and tracking of intraoperative hemorrhage and surgical instruments. The proposed common architecture comprises the location of the object of interest using feature texture, morphological information, and the tracking of the object based on Kalman filter for robustness with reduced error. The average recall and precision of the instrument detection in four prostate surgery videos were 96% and 86%, and the accuracy of the hemorrhage detection in two prostate surgery videos was 98%. Results demonstrate the robustness of the automatic intraoperative object detection and tracking which can be used to enhance the surgeon's preventive state recognition during robot-assisted surgery.

## 1. Introduction

Surgical robot technology has become a significant enhancement to laparoscopic surgery and ideally been suited for surgeries requiring minimal invasiveness and a high degree of dexterity [1–4]. However, current commercial surgical robot systems represented by da Vinci® (Intuitive Surgical, Inc., USA) have limitations such as limited vision through a laparoscope, absence of force or tactile feedback, and difficulties in agile tool maneuvering and exchange during emergency situations due to the bulky and complex configuration [5]. These limitations cause delayed perception and troubles in immediate responsive reconfiguration of the robot setting which may result in unsafe surgery. Two of representative surgical complications influenced by limited awareness of the surgical state are an acute hemorrhage and an instrument collision [6–8]. The use of additional monitoring techniques to facilitate faster awareness of the surgical state is particularly critical because an unexpected hemorrhage can occur due to the collision between the instrument and organs and lead to tissue perforation [6–8]. According to Blum et al. [9], uncontrollable bleeding from the cystic artery in cholecystectomy results in conversion from laparoscopic to open cholecystectomy. In addition to the complications, computerized endoscopic video analysis that provides new types of extra-anatomical information to surgeons can be beneficial for quantitative operation analysis of the surgery and the information archiving [4]. To contribute in endoscopic video analysis, we propose a novel method for object labeling technique using an efficient vision-based object classification algorithm framework that can be applied to various objects in the robot-assisted endoscopic surgery videos.

Although automatic object detection in endoscopic videos or other medical vision modalities has been studied in various applications, researches on common frameworks or universal algorithms for concurrent detection of multiple objects are limited. Furthermore, most studies pertaining to the application of vision techniques have focused only on the image-based instrument tracking methods which involve physical modification of the instruments mostly for analysis of the surgical workflow for skill evaluations during robot-assisted surgeries [10–17]. Conventional techniques regarding the surgical instrument position estimations utilize semantic information in images through segmentation technique [10, 16–18] or physical modifications in the surgical instruments which include attaching extra marker objects at the end of the instruments or adding color patterns at the tip of the instruments [19–22]. Recent methods include detections through convolutional neural network (CNN) [23, 24]. Given the training data sets of the surgical instrument images, the CNN processes and retrieves the presence and the location of the surgical instruments at the inference stage. However, the neural network algorithm is dependent on the training dataset. Additional considerations or training methods may be needed when an instrument with different shape that was not included in the training data or an object with shape changing over time such as hemorrhage is to be detected. The proposed algorithm utilizes only color properties of the object and can be applied to both rigid and deformable object, which we expect to provide a kind of common framework for the object detection and tracking in the surgical videos.

Tracking using the universal method proposed in this study can also offer better outcomes in terms of process performance than conventional methods when various objects should be detected during robot-assisted surgery. For higher performance of estimating positions in noisy and occluded environment, we chose to use a federated Kalman filter (FKF) [25], a filtering technique with a combination of a local and master filter, rather than using one Kalman filter. A combination of the proposed detection and tracking algorithms could act as a foundation for morphological change tracking of various objects in laparoscopic surgery videos.

The development of a machine vision system that enables robust segmentation and tracking of objects in laparoscopic images is a challenging task because the observed scenes are captured under time-varying lighting conditions and may have a moving background because of organ pulsation and breathing. In addition, a hemorrhage region can be temporarily occluded by the surrounded organs or the surgical instruments so that the region could be misidentified by the surgeons. To cope with difficulties in the processing of laparoscopic images that contain high speculation noise and nonuniform background, the proposed universal object tracking algorithm was structured with two characteristic stages: (1) feature extraction and (2) object tracking. In the first stage, features of objects for each sequential image are extracted using texture information and similarity measure, and the objects are tracked for the position optimally estimated by an improved Kalman filter in the second stage. Thus, we propose a surgical vision system using both the adaptive filter and the computer vision techniques to provide a reliable, fast, and robust detection and tracking which may enhance the robustness and the reliability of robotic laparoscopic surgery [13].

## 2. Materials and Methods

### 2.1. Algorithm Description

**Figure 1:**
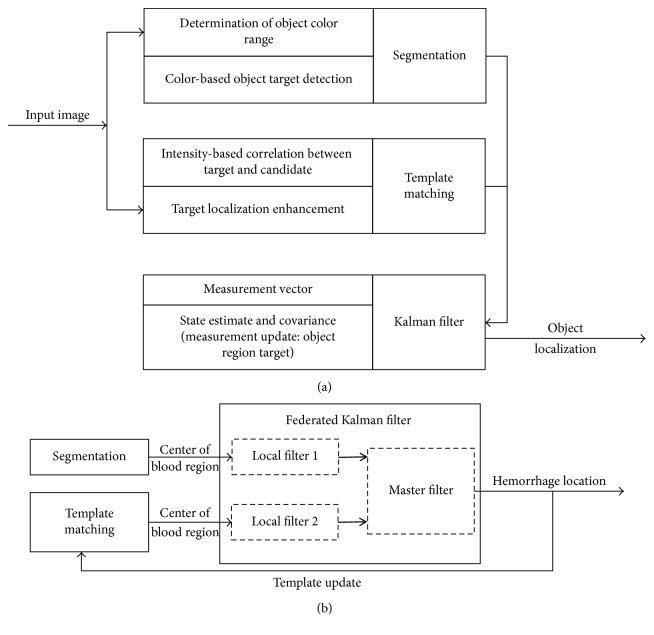
(a) Block diagram of the computational steps for various object detection including the structure of the proposed Kalman filter for optimal estimation. (b) FKF-based hemorrhage region tracking.

The method to identify objects in two-dimensional laparoscopic surgery images was based on local image features such as the texture and morphological properties of the objects.  Figure 1 shows the concept of the proposed method. Here, we identified the three main processing phases: (1) segmentation using specific texture properties of each image frame, (2) localization of the object using the correlation between image frames, and (3) optimal estimation of the current object locations using the Kalman filter.

In the first stage of the algorithm, the contrasted color signatures of the object were used to distinguish it from other environments so that the blood and the instruments could be differentiated from other organs. Under reasonable lighting conditions, the hemorrhagic blood had a distinct color signature different from other body tissues and the instruments, and the surgical instruments had metallic characteristics such as gray color. After processing images to enhance the textures of the blood and the instruments, the canny edge detection technique and the entropy filtering were thereafter applied to find a closed contour and decrease the background noise. Then, using binary edge detection, we were able to eliminate the irrelevant image details and maintain the shape features. Finally, the segmented boundaries of each blood region and the surgical instrument were marked on the original image.

If the objects to be detected are occluded by the noise or the detection was done under time-varying light conditions, the segmentation process might fail. To give robustness to the detection in such situations, the template-based matching algorithm that uses the average of previously identified regions for 0.5 s as a template was applied simultaneously. This method was strong to track the regions that might be neglected or falsely identified by methods using only local color features. It was expected that the combination of the color feature localization and the template matching would yield more accurate result than the use of only the former. To determine the best location of the test data that the samples match, a template-based approach that uses a sum-comparing metric, sum-of-squared difference (SSD), represented as

(1) $$\begin{array}{c} d\left(u,v\right)=\sum {\left(f\left(x,y\right)-t\left(x-u,y-v\right)\right)}^{2}, \end{array}$$

where *f*(·) denotes the original candidate image, *t*(·) denotes the template image provided after local color feature localization, and *x*, *y*, *u*, and *v* denote position parameters for the former and latter, respectively, was implemented. The minimum value of the SSD which elicited to be the highest correlated position was selected as the template matching algorithm in this paper. To improve the computation speed, we employed a calculation algorithm based on the fast Fourier transform (FFT) and downsized data by converting the spatial domain image into frequency domain signals.

### 2.2. Hemorrhage Recognition

In order to correctly distinguish a hemorrhage region from the environment during the segmentation process, the image contrast was enhanced using histogram equalization in each Red Green Blue (RGB) space. The problem due to similar color characteristics of the hemorrhage and organs was resolved by increasing the color contrast of the image. Using this contrasted image, a mutually inclusive threshold technique was implemented to identify the hemorrhage. Mutually inclusive algorithm is a semantic labeling technique, where different threshold is applied to each RGB space, and the positively labeled pixels that overlap in all RGB space are combined to form a target object mask image. Using this technique, blood regions were extracted.

At the final stage, followed by the template matching process, a federated Kalman filter (FKF) [25] was applied to the results of the segmentation and template matching processes to estimate the location of the hemorrhage region. The FKF, consisting of two local filters and one master filter, was a model-based method to estimate the measurement data containing noise and other inaccuracies. The filtering technique distributed estimation problems through a local and a master filter and hence reduced the calculation load and provided independent faulty measurement detection [25]. The first part of the FKF, an estimator from the local filters that were applied to segmentation and template matching, was implemented to detect and reduce tracking outliers during the operation. The outputs from the local filters were then applied to the master filter for tracking the final location of the hemorrhage region as shown in Figure 1.

### 2.3. Surgical Instrument Recognition

Before the segmentation process, instruments and organs were classified into different categories using k-means clustering under LAB space, where L stands for luminance and A and B for two color channels. The k-means clustering technique was used to classify the different objects based on the image intensity variances. The best results have been provided under LAB space because of its advantages for the image sharpening. Histogram equalization applied in the hemorrhage detection was not needed in the surgical instrument detection because the instruments and the organs could be easily differentiated by the intensity characteristics. After a chain of image processing techniques, the output was combined with the results from the instrument motion subtraction, which is the movement computed by subtracting interframe images. Finally, each instrument was labeled.

After completing the segmentation stage, the numbered labels were assigned to the instruments as each was first detected. Once an instrument was identified, the center location of the instrument on the image was saved and compared with the result by the Kalman filter algorithm in the final stage.

The Kalman filter technique was used to compensate for the identification or the tracking failures due to the occlusion. When the both segmentation and the template matching failed to detect the instrument correctly, the Kalman filter functioned as an estimator of the instrument position. The gain was continually adjusted with respect to the calculated Euclidean distance between the center points of the instrument in the current and the previous image frames. The instruments were given their lifetime when they were first detected. The continual recognition of instruments increases their lifetime by 20%. When the instruments vanished and could not be recognized, the lifetime was decreased. The lifetime of 0% stated that the instrument did “not exist.” In addition, the distances of each labeled location and their previous locations for the instrument were calculated as the Euclidean distance to find the correct label. The proposed FKF method provided more accurate detection and reduced the tracking failures by continuously adjusting gains of the master Kalman filter built on the prior knowledge acquired from each stage of segmentation and template matching, than without filtering.

## 3. Results

For this study, four videos for the laparoscopic surgical operation with resolutions of 640 × 480 pixels and 10 frames/s in MPEG format were used. Each video of approximately 20 s consisted of a sequence of the different surgical tasks under different lighting conditions, and two of them contained the hemorrhage events. The processing and the analysis steps were implemented using MATLAB (MathWorks, Natick, MA, USA). The identified object region was marked at its geometric center, indicating the median of the detected boundary pixels, and the hemorrhage area was measured by counting the pixels in the entire hemorrhage region. The ground truth data sets for the hemorrhage were prepared by manually inspecting and marking the hemorrhage region, and for the surgical instruments, the centers were calculated by marking their boundaries in each image. The ground truth data were prepared by a beginner medical analyst and confirmed by an expert surgeon. To evaluate the performance of the proposed method, recall and precision were measured:

(2) $$\begin{array}{c} \begin{array}{l} \mathrm{Recall}=\frac{\mathrm{TP}}{\mathrm{TP}+\mathrm{FN}},\\ \mathrm{Precision}=\frac{\mathrm{TP}}{\mathrm{TP}+\mathrm{FP}}. \end{array} \end{array}$$

True positive (TP) was counted when the distance of centroids between the detected object and the ground truth was less than 1/15 of an image size 640 × 480, which corresponded to average 42 mm × 25 mm in the physical unit. Since the surgical instrument size normally occupied 1/8 of an image, the centroid distance of 1/15, approximately 2.8 mm in the physical unit, was reasonable. False negative (FN) refers to the missing object where the object was not detected, but the ground truth object was present. False positive (FP) was a false alarm where an object was detected, but no ground truth objects was present. FP was also counted when the centroid distance difference between the detected object and the ground truth was over 1/15 of an image size.

The distance error, root mean square error (RMSE), between the estimated and the reference centroids for the hemorrhage region and the surgical instrument were computed as in (3), where ${\left[\hat{x}\left(i\right),\hat{y}\left(i\right)\right]}^{T}$ denotes the estimated center position, [*x*_r_(*i*), *y*_r_(*i*)]^*T*^ denotes the reference center position, and *N* denotes the number of hemorrhage. The blood flow was also analyzed with the area calculated by the number of the pixels inside the boundary of the detected blood region in each frame:

(3) $$\begin{array}{c} \mathrm{RMSE}=\sqrt{\frac{1}{N}\sum_{i=1}^{N}\left\{{\left(\hat{x}\left(i\right)-x_{r}\left(i\right)\right)}^{2}+{\left(\hat{y}\left(i\right)-y_{r}\left(i\right)\right)}^{2}\right\}}. \end{array}$$

### 3.1. Hemorrhage Recognition

The average RMSE values over the entire time period for the segmentation-only method, the output of the segmentation stage where the semantic information of an image was used to extract the hemorrhage region, and the proposed method were 5.6 and 3.6 pixels, and the accuracy were 87% and 98%. The accuracy refers to counting the presence of the hemorrhage frames out of total frames in a video.

**Figure 2:**
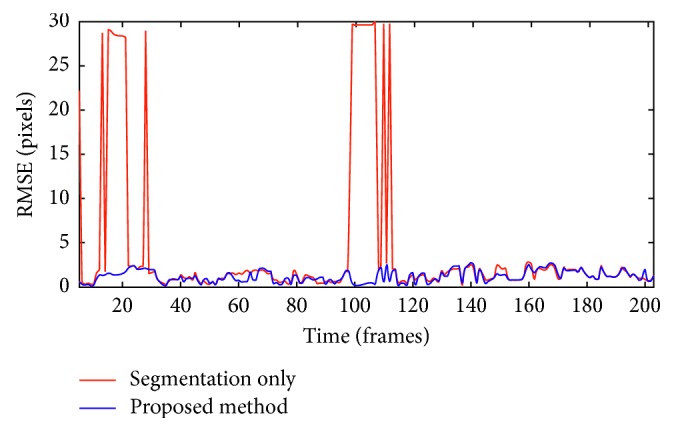
RMSE profiles of the proposed method and the segmentation-only method. In both cases, centroids of the manually traced bleeding region were used as ground truth values.

Figure 2 displays the RMSE profiles for the proposed method and the segmentation-only one for an approximately 20 s time period. Between frame numbers 100 and 120, the segmentation-only method was unable to detect the blood region that was temporarily hidden by obstructing objects, resulting in maximum RMSE values. Moreover, the average computation time per frame was 0.89 s, which is adequate for processing the hemorrhage detection in reality when the hemorrhage does not flow massively within 10 frames, as shown in Figure 2.

**Figure 3:**
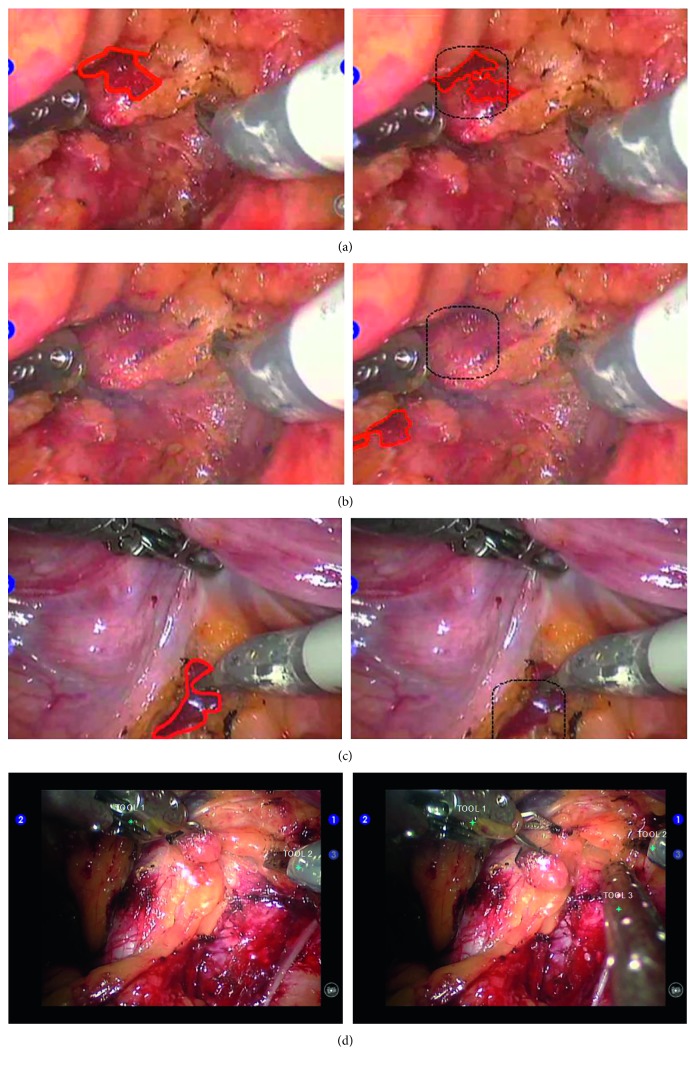
Selected frames of hemorrhage region detection and instrument detection results. In (a), (b), and (c), the left figures display the manual detection of the hemorrhage, and the right figures display the automatic detection by the proposed method: (a) both the segmentation-only method (red boundary) and the proposed technique (square mark) correctly detected the hemorrhage region; (b) manually undetectable hemorrhage has been detected accurately by the proposed technique using the previous frame information, but falsely segmented by the segmentation-only method; (c) the hemorrhage was not detected by the segmentation-only method, but accurately detected by the proposed method. (d) Sample frames of multiple instrument detection.

Figure 3 shows that the FKF in the proposed method produced a more stable RMSE profile even during the occlusion period. As a result, the proposed method yielded lower tracking errors as well as robustness to occlusion.

### 3.2. Hemorrhage Flow Analysis

**Figure 4:**
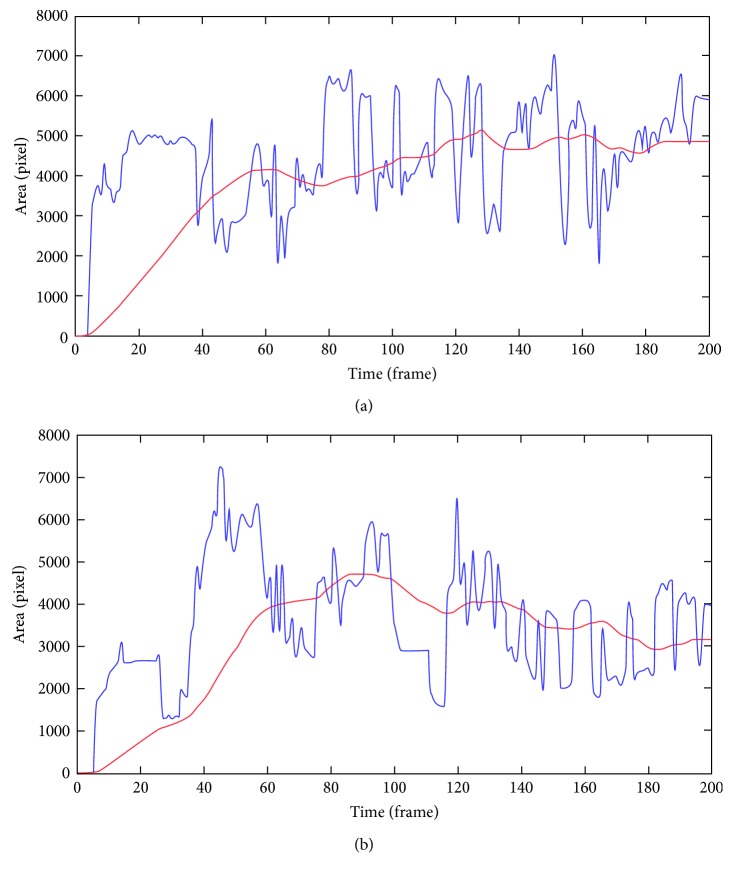
Hemorrhage flow classification by area variation analysis where the red line indicates linearity of the hemorrhage flow and the blue line indicates the calculated the hemorrhage area: (a) stagnant hemorrhage; (b) hemorrhage flow following stanch.

Figure 4 shows the area variation analysis that classifies types of the hemorrhage into flowing or stagnant. The overall scheme is provided by the red line, which is plotted by calculation of peaks of area increase and decrease. After several frames, this operation can specify dangerous targets by warning signs. Figure 4 shows a stagnant hemorrhage with a very slow increase in blood volume due to insufficient stanch operation. Other than frames 40 to 80 where the blood area was reduced due to smoke and occlusions, the overall peak area tended to keep at its level. Due to the little variation, this can be considered as nonpersistent bleeding and thus classified as a nonwarning target. Figure 4 shows an acute hemorrhage that rapidly flows around the surgical view, which was controlled by the surgeons after the situation awareness. The fast increase in the first 50 frames describes rapid blood flow due to an unintended agitation. The linear decrease after frame 60 shows that the hemorrhage was treated and removed by the surgical tools such as forceps and suction. As interpreted in Figure 4, the calculated area analysis depicts the surgical events that occur during the surgery. Therefore, the blood-flow profile can be used in actual surgery for surgeons to be warned of the state of the blood flow and control blood flow rapidly before they become aware of the situation.

### 3.3. Surgical Instrument Recognition

**Figure 5:**
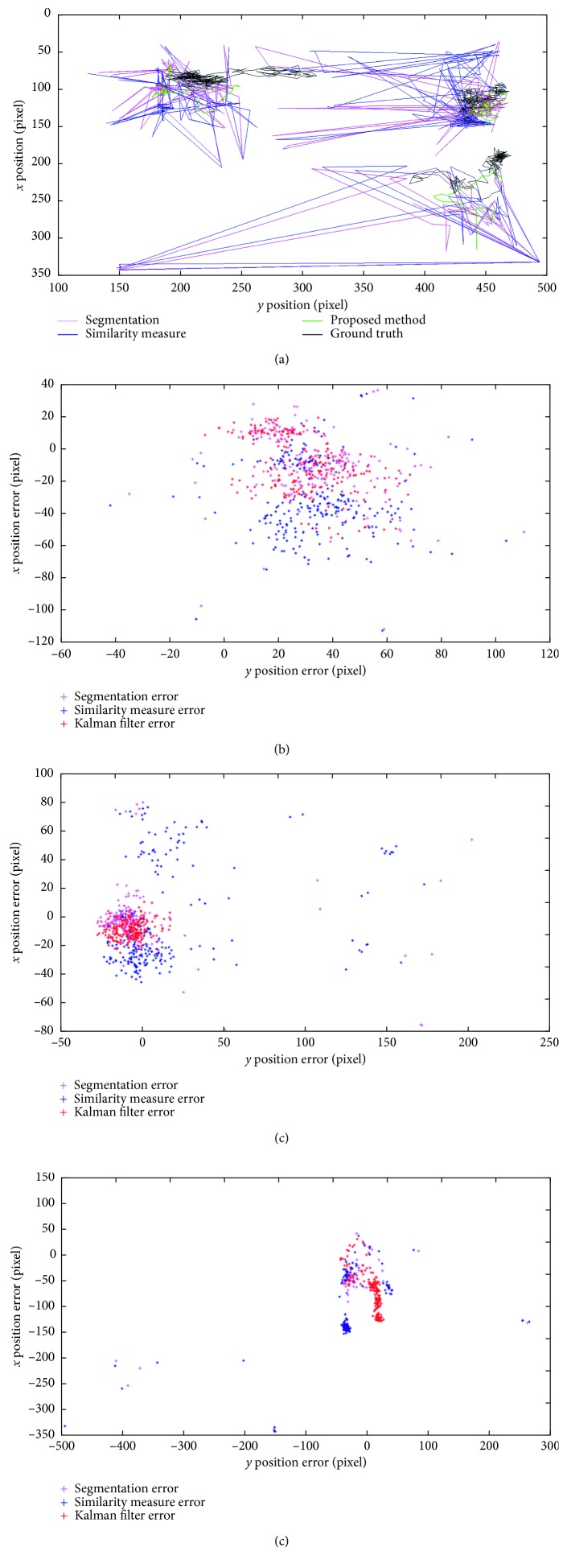
Instrument tracking: (a) the proposed method validation using multiple instrument path trajectories in comparison with manually traced values, *x* and *y* pixel positions of instruments 1, 2, and 3; (b) path position distance error of instrument 1; (c) path position distance error of instrument 2; (d) path position distance error of instrument 3.

The instrument trajectories using the three different position identification methods—one with only segmentation, one with the similarity measure analysis added to the former, and the proposed one with the additional Kalman filter—were calculated in the test video and are shown in Figure 5. As the figure depicts, the proposed method was the closest to the identified ground truth value. The proposed method outperformed the other two methods which contained noise peaks and failed to accurately detect the instrument locations. The result of the proposed method was also quantified through the recall and precision measurements, where each surgical instrument detection rate was 96% and 86% in the four robot-assisted laparoscopic videos. Additionally, the RMSE analysis for the instrument 1, 2, and 3 resulted in 39, 15, and 74 pixels, respectively. The average RMSE of 42 pixels was approximately 1/16 of the image size, about 2.6 mm in the physical unit, which was sufficient for the instrument localization where the instrument usually took 1/8 of the image size. The instrument tracking system on a laparoscopic surgery video was implemented as shown in Figure 3.

## 4. Discussion

Surgeons often face difficulty in environmental perception during robot-assisted surgery, and this may indirectly lead to delayed maneuvering of the tools [8]. Despite the importance of the timely detection and the management of the incidental hemorrhage, the automatic recognition and the localization of objects during laparoscopic surgery have not yet been widely and fully studied. Most existing object recognition methods work only with the defined objects. To elaborate, the algorithms that are studied to detect the surgical instruments do not normally work for the hemorrhage detection. For example, the neural network algorithm would work only on the defined objects that have been trained, and the conventional techniques that were used for the surgical instrument detection focus only on the rigid objects. With our proposed algorithm, deformable object such as the hemorrhage could also be detected because we consider texture rather than the shape in object detection. Thus, we developed a novel algorithm to detect and track both the hemorrhage and the surgical instruments in laparoscopic video images taken during real robot-assisted surgery. By extending our method, extra information and control such as organ tracking could also be obtained.

The performance of the algorithm was evaluated with the actual video data from a surgery. For the hemorrhage detection, the segmentation-only method provided the average RMSE values of 5.6 pixels, which is about 1.5% of the image size. The percentage means that the distance error was considerably small. However, this error was further reduced to 3.6 pixels when the proposed method was applied. The measurement of flow of the hemorrhage also demonstrated its effectiveness by depicting the surgical situations, which displayed a linear increase when blood was flowing and a decrease when the hemorrhage was stanched. From our inferenced surgical videos, the average computation time was approximately 1.1 Hz, which is sufficient for the hemorrhage detection and the fast state recognition since the blood does not flow massively within 1 s. The process time can also be decreased through an improvement in software programming and hardware with higher computation speed.

**Figure 6:**
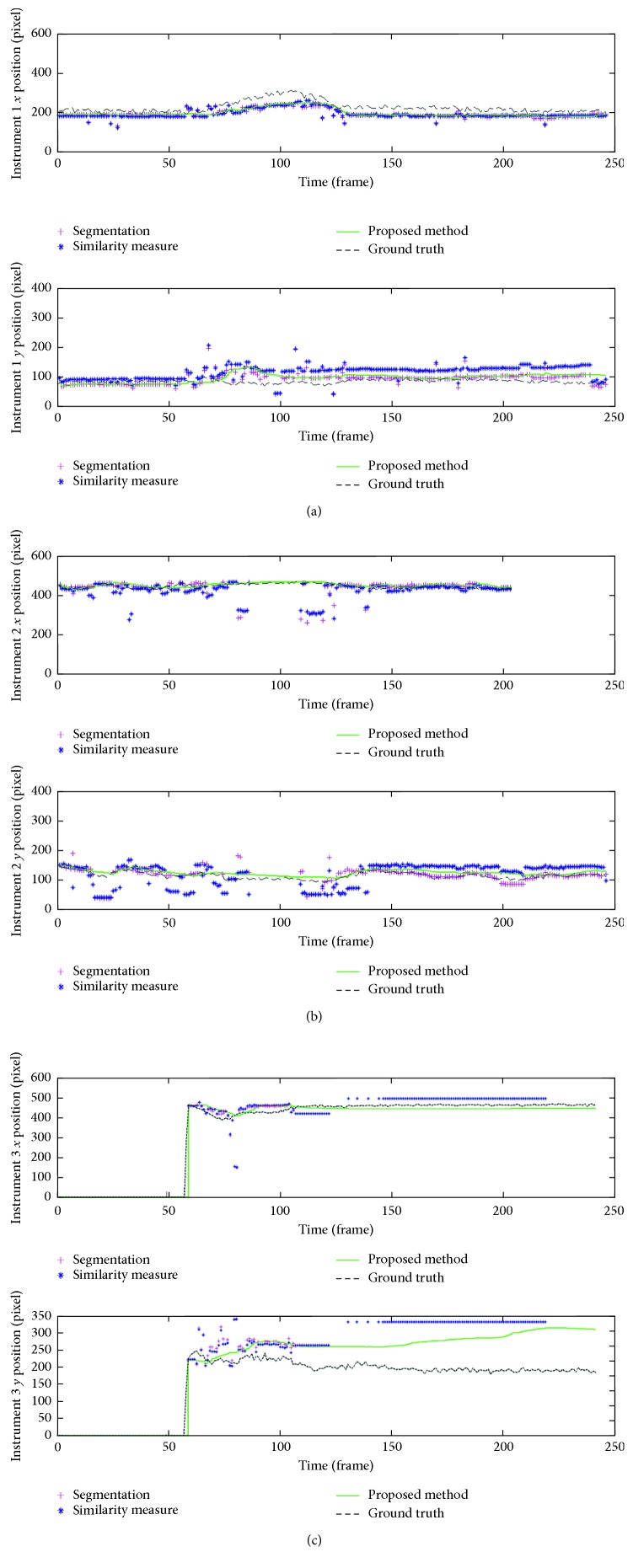
Validation of the surgical instrument position trajectories in time domain. The plot shows *x* and *y* position of the (a) instrument 1, (b) instrument 2, and (c) instrument 3 as reported by manually traced values (dashed line) and the proposed tracking algorithm (solid line).

In addition to the hemorrhage detection, the surgical instrument recognition made simultaneous multiple instrument tracking without additional hardware feasible and resulted in the recognition rate of over 80%. Due to test dataset differences, recent works with the neural network instrument detection [24] and our algorithm cannot directly be compared. However, our algorithm showed higher extension of implementation and higher precision than that of Choi et al. [24] which resulted in mean average precision (mAP) of 72.26% and was limited to 8 instruments. One limitation in the current implementation is the dependency of segmentation accuracy on instrument color and surface texture characteristics. As depicted in Figures 5 and 6, the irrigation instrument with holes on the surface is prone to relatively higher identification error; however, it might be less likely to cause accidental injuries such as grabbing or cutting tissues, compared to other instruments.

Overall, the proposed method provided more accurate detection results using mathematical filters built on knowledge acquired from previous object locations. It outperformed the segmentation-only technique, which fails to accurately detect bleeding and instrument locations mainly because of environmental distortions such as smoke, camera motion, or organ occlusion. The analytic tools such as RMSE and area measurement showed that the proposed method can provide surgeons with information about object movement and unsafe situations that may occur during surgery.

## 5. Conclusion

In a complicated environment such as that in a robot-assisted laparoscopic surgery, in which the surgeon experiences limited vision through a laparoscope, the proposed automatic object recognition function will help surgeons rapidly handle emergency situations through fast robot arm control. The system using the method can be used as a concept to further extend to warn the surgeon if the hemorrhage flow occurs out of sight due to the movement of the camera or if the surgical instruments are about to collide. Also, using the concept of localizing the center points of the object, the endoscopic camera may be able to automatically reach the focused surgical site.

With the proposed method, additional functionality to increase the safety of robotic control and the surgical procedure was implemented without additional hardware such as extra artificial markers, specialized instruments, or separated cameras or detectors. Warnings of the surgical unsafety through the automatic detection of bleeding and instrument positions will provide useful information to surgeons so that they can perform safer surgeries and reduce overall surgery time. This method will also be extended to other surgical state recognition applications in which spatial and temporal accuracies of feature location are critical.
